# Low-fat diet, and medium-fat diets containing coconut oil and soybean oil exert different metabolic effects in untrained and treadmill-trained mice

**DOI:** 10.1186/s12970-018-0234-y

**Published:** 2018-06-18

**Authors:** Mark Christian Manio, Shigenobu Matsumura, Kazuo Inoue

**Affiliations:** Department of Food Science and Biotechnology, Graduate School of Agriculture, Kyoto University, Kyoto, Japan

**Keywords:** Low-fat diet, Soybean oil, Coconut oil, Treadmill exercise, Endurance, Glycogen, Mitochondria, Metabolism, Training-adaptation

## Abstract

**Background:**

Diets containing fats of different proportions and types have been demonstrated to influence metabolism. These fats differ in long chain fatty acids (LCFAs) or medium chain fatty acids (MCFAs) content. In our laboratory using swimming as the training modality, MCFAs increased endurance attributed to increased activities of oxidative enzymes. How it affects whole-body metabolism remains unexplored. The present study investigated the metabolic, biochemical and genetic adaptations with treadmill running as the training modality.

**Methods:**

C57BL/6N mice were divided into untrained and trained groups and provided with low-fat (10% kcal from soybean oil), coconut oil (10% kcal from soybean oil, 20% kcal from coconut oil) or soybean oil (30% kcal from soybean oil) diet. Training was performed on a treadmill for 30 days. After recovery, whole-body metabolism at rest and during exercise, endurance, substrate metabolism, mitochondrial enzyme activities, and gene expression of training-adaptive genes in the muscle and liver were measured.

**Results:**

At rest, medium-fat diets decreased respiratory exchange ratio (RER) (*p <* 0.05). Training increased RER in all diet groups without affecting oxygen consumption (*p <* 0.05). During exercise, diets had no overt effects on metabolism while training decreased oxygen consumption indicating decreased energy expenditure (*p <* 0.05). Coconut oil without training improved endurance based on work (*p <* 0.05). Training improved all endurance parameters without overt effects of diet (*p <* 0.05). Moreover, training increased the activities of mitochondrial enzymes likely related to the increased expression of estrogen related receptor (ERR) α and ERRβ (*p <* 0.05). Coconut oil inhibited peroxisome proliferator-activated receptor (PPAR) β/δ activation and glycogen accumulation in the muscle but activated PPARα in the liver in the trained state (*p <* 0.05). Substrate utilization data suggested that coconut oil and/or resulting ketone bodies spared glycogen utilization in the trained muscle during exercise thereby preserving endurance.

**Conclusion:**

Our data demonstrated the various roles of diet and fat types in training adaptation. Diets exerted different roles in PPAR activation and substrate handling in the context of endurance exercise training. However, the role of fat types in training adaptations is limited as training overwhelms and normalizes the effects of diet in the untrained state particularly on endurance performance, mitochondrial biogenesis, and ERR expression.

## Background

Oils from different plant sources differ in fatty acid composition. Coconut oil is rich in lauric acid (C12:0), comprising about 45–53% of the total fatty acid composition, while having very low content of fatty acids above C14 [1–3]. Soybean oil, on the other hand, is rich in polyunsaturated and monounsaturated fatty acids particularly linolenic acid (C18:3), linoleic acid (C18:2) and oleic acid (C18:1) occupying 7.8, 53.2 and 23.4%, respectively of its total fatty acid composition while almost absent of C14 fatty acids and below [3, 4]. Differences in fatty acid content and composition not only lead to diverging physico-chemical properties but also metabolic fates. Medium chain fatty acids (MCFAs; C8-C14) can enter cells without requiring fatty acid transporters unlike long chain fatty acids (LCFAs; C16-C22) [5]. However, both types require carnitine activation in the muscle mitochondria [5]. Moreover, most MCFAs are absorbed and metabolized in the liver for conversion to ketone bodies or incorporation to liver triglycerides (TG) especially with prolonged feeding [6, 7]. These observations imply that MCFAs function as direct or precursor energy substrates for non-hepatic organs.

Training leads to several adaptations in whole body and organs. In the muscle, prolonged training increases glycogen storage, fatty acid uptake and utilization, and mitochondrial biogenesis among others [8]. Trained muscle is then able to produce energy to sustain endurance. These adaptations are brought about by contraction- and high AMP/ATP ratio-induced AMP-activated protein kinase (AMPK) activation accompanying prolonged training [8]. The peroxisome proliferator-activated receptors (PPARs) family and the estrogen-related receptors (ERRs) family are involved in these transcriptional adaptations with the coactivator PPARγ-coactivator 1α (PGC1A) synchronizing signals from AMPK and therefore of exercise [9].

Fats with varying fatty acid composition differentially affect PPAR isotypes. PPARα and PPARβ/δ are highly expressed in peripheral tissues such as the muscle and control genes for oxidative metabolism while PPARγ is present mainly in adipose tissues orchestrating adipogenesis [10]. LCFAs activate PPARs while the intensity of activation is influenced by the type of fatty acid and the PPAR isotype [11, 12]. MCFAs, particularly C10 and C12 strongly activate PPARγ while C10 and C14 activate PPARα and PPARβ/δ to a certain extent [11–14]. Also, MCFAs upregulate mitochondrial biogenesis better than LCFAs [15]. Therefore, MCFAs and LCFAs function as direct or indirect signaling molecules for mitochondrial oxidative capacity possibly through PPARα and/or PPARβ/δ [9, 15–17]. Unlike PPARs, no endogenous ligands for ERRs have been identified. However, ERR isotypes are constitutively active and the binding of PGC1A potentiates their activity [18, 19]. Therefore, downstream effects of training on transcriptional adaptations are also influenced not only by the expression of these transcription factors but also by available fatty acids for activation.

The effects of short- and long-term feeding of MCFAs and LCFAs with or without training have been investigated. In our laboratory, MCFAs (coconut oil) improved swimming capacity in trained mice and increased mitochondrial enzyme activities relative to LCFAs (soybean oil) [20]. With training, MCFAs (C8 and C10) increased energy expenditure in rats relative to LCFAs (rapeseed oil) [21]. In untrained mice, increased mitochondrial markers with MCFAs (coconut oil) relative to LCFAs (lard) was associated with whole body and localized muscle oxygen consumption as assessed with in vitro, ex vivo and in vivo experiments [15]. In rats, while MCFAs (C8 to C12) did not affect endurance performance, LCFAs (primarily C16) decreased endurance associated with increased cardiac mitochondrial uncoupling [22]. In contrast, LCFAs (soybean oil) with training did not impair but rather improved endurance on a treadmill in wild-type C57BL/6J mice [23]. Despite these previous investigations, literature on the comparative effects of MCFAs and LCFAs on training adaptations remains scarce. Furthermore, the absence of comparison with high-carbohydrate/low-fat diet may limit the adaptability of these diets in athletic dietary management.

The objective of the study was to update the current knowledge of physiological adaptations occurring during exercise training with low-fat diet, and medium-fat diets containing coconut oil or soybean oil specifically on whole-body metabolism at rest and during exercise, substrate metabolism, mitochondrial functions, and genetic adaptive responses in the muscle and liver under the treadmill exercise modality.

## Methods

### Animals

Seven (7)-week old male C57BL/6N mice were purchased from Shimizu Laboratory Supplies (Hamamatsu, Shizuoka, Japan). Mice were housed in an animal room at 22 ± 0.5 °C and 50% humidity with a 12 h light-dark cycle (lights on and off at 6:00 and 18:00, respectively). Mice were acclimatized to this environment with ad libitum access to a standard chow diet (Oriental Yeast Co., Tokyo, Japan) and water for 7 to 10d before changing to assigned diets. Mice were randomly assigned to the following purified diets: low-fat diet (L; 20% kcal from casein, 70% kcal from cornstarch and 10% kcal from soybean oil); coconut oil diet (C; 20% kcal from casein, 50% kcal from cornstarch, 10% kcal from soybean oil and 20% kcal from coconut oil); and soybean oil diet (S; 20% kcal from casein, 50% kcal from cornstarch and 30% kcal from soybean oil). All diets contained vitamin and mineral mixes and were prepared by Research Diets (NJ, USA) based on the D12450K formulation. Training of mice was commenced following diet assignment. Dietary groups were divided into untrained (U) and trained (T) groups. Animal experiments were performed according to the Kyoto University Guidelines for the Ethical Treatment of Laboratory Animals as approved by the Kyoto University Animal Experimentation Committee with the number (29–39).

### Treadmill training

Training was conducted daily for 30d from 6:00 using a treadmill for rodents (MK-680; Muromachi, Tokyo, Japan). On the first 15d, mice ran for 1 h at 15 m/min at 3° incline. From the 16th day, intensity was increased to 18 m/min. Mice were forced to run by poking. All mice completed the training program.

### Basal indirect calorimetry

Basal indirect calorimetry using the ARCO-2000 system (Tokyo, Japan) under ad libitum feeding and resting conditions as described in [24] was performed on a subset of mice. Mice were assigned to calorimetry chambers on the 28th day of training to facilitate acclimatization. Actual measurement was performed 24 h to 48 h post-training which represents a full light-dark cycle devoid of acute exercise effects. Mice were sacrificed after measurements. Sample collection was based on Manio, et al. [23].

### Exercise indirect calorimetry and endurance test

Indirect calorimetry during exercise with treadmill endurance test was performed on a subset of mice. About 1.5-2 h before the run (6:00), mice were placed in sealed treadmill chambers (Mousebelt-200; Arco System, Tokyo, Japan) at 10° incline to acclimatize. After warming-up for 2 min, intensity was increased to 15 m/min. After 30 min, the intensity was increased to 18 m/min and maintained for 30 min. Then, the intensity was increased to 21 m/min and kept herein until exhaustion. Mice were stimulated with electrical stimulus (0.2 mA) and occasional noise and poking. Exhaustion was ruled if mice remained on electrodes or could not sustain running for 20s despite additional stimulation.

### Fixed time run

Fixed time run was performed on a subset of mice. Food was removed 2 h before running. Mice were individually placed on a moving treadmill set at an intensity of 10 m/min. After 2 min of warm-up, mice were transferred to a moving treadmill set at 15 m/min, 10° incline. After exactly 30 min, mice were removed from the treadmill and immediately sacrificed. Experiments were performed in the same conditions as exercise indirect calorimetry.

### Blood chemistry and tissue metabolites

Serum was measured for glucose, triglycerides (TG), non-esterified fatty acids (NEFA) and beta-hydroxybutyrate (β-HB). Glycogen and TG were measured in organs [25–27]. Measurements are detailed in [23].

### Protein extraction and enzyme activities

Muscle and liver were lysed in a 1% NP-40 buffer as detailed in [23]. Beta-hydroxyacyl-CoA dehydrogenase (β-HAD) activity was measured according to a procedure by Holloway, et al. [28]. Succinyl-CoA:3-oxoacid CoA-transferase (SCOT) activity was measured based on Williamson, et al. [29]. Citrate synthase (CS) activity was measured according to Srere [30]. Cytochrome c oxidase of the mitochondrial electron transport chain, also known and hereby referred to as Complex IV, was measured based on Mac Arthur, et al. and Spinazzi, et al. [31, 32]. Acetoacetyl-CoA thiolase (AACT) and deacylase (AACD) activities were measured based on Williamson, et al. [33]. All enzyme activity measurements were modified to adapt to a 96-well plate system as detailed in [23].

### Reverse transcriptase quantitative polymerase chain reaction (RT-qPCR)

Total RNA was extracted with Tripure Isolation Reagent (Roche, Mannheim, Germany) and GenElute Mammalian Total RNA Miniprep Kit (Sigma-Aldrich, MO, USA) as detailed in [23]. Total RNA (1.8 μg and 1.5 μg for muscle and liver, respectively) was reverse transcribed with Transcriptor First Strand cDNA Synthesis Kit (Roche, Mannheim, Germany). Messenger RNA (mRNA) expression levels were quantified using intron-spanning primers and corresponding Universal Library Probes (Roche, Mannheim, Germany) listed in Table 1. Values were rationalized to *Hprt* expression [34].

**Table 1:** Primers and probes in RT-qPCR

Gene name	Sequence (5′ to 3′)	Universal Probe No.	Accession No.
PPARγ Coactivator 1α, (Pgc1a)	F: tgtggaactctctggaactgc	63	NM_008904.2
	R: agggttatcttggttggcttta		
PPARα, (Ppara)	F: ccgagggctctgtcatca	11	NM_011144.6
	R: gggcagctgactgaggaa		
PPARβ/δ, (Pparb/d)	F: atgggggaccagaacacac	11	NM_011145.3
	R: ggaggaattctgggagaggt		
ERRα, (Erra)	F: gtgggcatgctcaaggag	29	NM_007953.2
	R: ggaaaggcaaagggtcca		
ERRβ, (Errb)	F: ggcgttcttcaagagaacca	49	NM_011934.4
	R: tccgtttggtgatctcacatt		
ERRγ, (Errg)	F: aagtgggcatgctgaaagaa	29	NM_011935.3
	R: cagcatctattctgcgcttg		
Lipoprotein lipase, (Lpl)	F: tggataagcgactcctacttcag	22	NM_008509.2
	R: tccctagcacagaagatgacc		
Carnitine palmitoyltransferase 1B, (Cpt1b)	F: ccatcattgggcacctct	104	NM_009948.2
	R: gtctccgtgtagcccaggt		
Glucose transporter, 4 (Glut4)	F: tcgtcattggcattctggt	104	NM_009204.2
	R: agcagtggccacagggta		
Fatty acid transport protein, 1 (Fatp1)	F: cttcctaaggctgccattgt	49	NM_011977.3
	R: ggcagtcatagagcacatcg		
Myosin heavy chain, 2a (Myh2)	F: tcttctctggggcacaaact	22	NM_001039545.2
	R: cccttcttcttggcaccttt		
Carnitine palmitoyltransferase, 1A (Cpt1a)	F: aaagcaccagcacctgtacc	34	NM_013495.2
	R: aacctccatggctcagacag		
3-Hydroxy-3-methylglutaryl-CoA synthase, 2 (Hmgcs2)	F: ctgtggcaatgctgatcg	93	NM_008256.4
	R: tccatgtgagttcccctca		
Hypoxanthine-guanine phosphoribosyl transferase, (Hprt)	F: cctcctcagaccgcttttt	95	NM_013556.2
	R: aacctggttcatcatcgctaa		

F: forward

R: reverse

### Immunoblotting

Protein concentration of lysates was adjusted with lysis buffer and 4× Laemilli buffer containing 20% mercaptoethanol. Samples were loaded on 8% polyacrylamide gels. Semi-dry transfer to PVDF membranes was performed in a transfer buffer containing 20% methanol. After transfer, Ponceau S staining was performed. Excess stain was removed in distilled water. Membranes were visualized and digitized (LAS-3000; Fujifilm, Tokyo, Japan). Membranes were blocked in 5% skim milk powder in Tris-buffered saline with 0.1% Tween-20 buffer (TBST) containing 0.05% ProClin 300 (Sigma Aldrich, MO, USA). Membranes were cut at sections corresponding to regions of molecular weight previously identified to contain the protein of interest. Membranes were incubated in goat anti-fatty acid translocase/cluster of differentiation 36 (CD36) antibody (1:2000; AF2519, R&D Systems, MN, USA), goat anti-pyruvate dehydrogenase kinase 4 (PDK4) antibody (1:1000; C-16, Santa Cruz Biotechnology, CA, USA), rabbit anti-PGC1A antibody (1:1000; H-300, Santa Cruz Biotechnology, CA, USA) or rabbit anti-glucose transporter 4 (GLUT4) antibody (1:2000, AB1346, Chemicon International, CA, USA) at 4 °C for 20 h. Membranes were washed 3× in TBST to remove excess primary antibody. Horseradish peroxidase-labelled secondary antibody incubation was performed in anti-goat IgG (1:1000; P0449; Dako, Tokyo, Japan) or anti-rabbit IgG (1:1000; P0399; Dako, Tokyo, Japan) for 3 h at 4 °C. After washing 3× in TBST to remove excess secondary antibody, membranes were visualized by chemiluminescent detection (Western Lightning Plus ECL; Perkin Elmer, MA, USA). Signals corresponding to PGC1A (91 kDa), CD36 (88 kDa), GLUT4 (58 kDa), PDK4 (47 kDa) and Ponceau S signals were quantified using the software MultiGauge V3.2 (Fujifilm, Tokyo, Japan) with automatic background detection.

### Statistical analyses

Statistical analyses were performed using the Prism 5.0 software (Graphpad Software, CA, USA). Points in exercise indirect calorimetry time-course data are presented as means ± SEM. Each time point was analyzed by one-way analysis of variance (ANOVA) to compare groups of different diets and by Student’s unpaired t-test to compare groups of different training states receiving the same diet. Other data are presented as means ± SEM and were analyzed accordingly using one-way ANOVA followed by Newman-Keuls post-hoc test and Student’s t-test. Significance level (α) was set at 0.05.

## Results

### Training increased calorie intake in low-fat diet but did not affect body weight

Food intake was measured every third day. Total food and calorie intake for 30 days were calculated (Table 2). Body weight and weight of some organs were measured. Total food intake decreased in C and S relative to L in either training state (*p* < 0.05). Training did not increase food intake in C and S but significant increase was observed in L (*p* < 0.05). Total calorie intake showed no difference among diet groups in either training state. Training increased calorie intake in L (*p* < 0.05). Despite this, diets nor training did not cause significant body weight change at the end of the experimental period relative to day 1. While significant differences were not observed in muscle and fat weight among diet groups of either training state, training significantly increased liver weight in L and S (*p* < 0.05) but not in C. Therefore, the type of diet combined with training affected calorie intake and liver weight but not body weight.

**Table 2:** Body and organ weights, and food intake

Training		Untrained			Trained		
Diets		Low-fat	Coconut oil	Soybean oil	Low-fat	Coconut oil	Soybean oil
Food intake, g (*n* = 5–9)		91.26 ± 0.86^a^	84.66 ± 0.95^b^	84.00 ± 1.72^b^	96.81 ± 2.66^a^*	86.82 ± 1.73^b^	82.97 ± 1.34^b^
Calorie intake, kcal (*n* = 5–9)		351.4 ± 3.3	364.1 ± 4.1	361.2 ± 7.4	371.7 ± 10.2*	373.3 ± 7.4	356.8 ± 5.8
Body weight (*n* = 24–25)	D1, g	22.93 ± 0.22	23.02 ± 0.27	22.94 ± 0.25	23.01 ± 0.23	23.02 ± 0.19	22.84 ± 0.27
	D32, g	25.92 ± 0.37	26.86 ± 0.45	26.12 ± 0.32	26.99 ± 0.38	27.42 ± 0.37	26.47 ± 0.40
	%∆	13.04 ± 1.18	16.84 ± 1.88	13.97 ± 1.35	17.56 ± 2.08	19.35 ± 2.03	15.64 ± 2.17
Gastrocnemii, g (*n* = 8)		0.247 ± 0.005	0.243 ± 0.006	0.244 ± 0.006	0.254 ± 0.008	0.252 ± 0.008	0.245 ± 0.006
Liver, g (*n* = 8)		1.289 ± 0.069	1.22 ± 0.070	1.104 ± 0.027	1.491 ± 0.060*	1.306 ± 0.047	1.322 ± 0.061*
Epididymal fat, g (*n* = 8)		0.667 ± 0.056	0.754 ± 0.058	0.590 ± 0.049	0.587 ± 0.032	0.660 ± 0.039	0.686 ± 0.038

D1: day 1

D32: day 32

%∆: %change at day 32 from day 1

^a,b^: dissimilar alphabets indicate significant difference (*p* < 0.05) among groups of the same training status as assessed by one-way ANOVA followed by Newman-Keuls post-hoc test

*: *p <* 0.05 vs untrained counterpart as assessed by Student’s t-test

### Coconut and soybean oil decreased basal respiratory exchange ratio (RER) without affecting oxygen consumption (VO_2_) in untrained and trained states

**Fig. 1:**
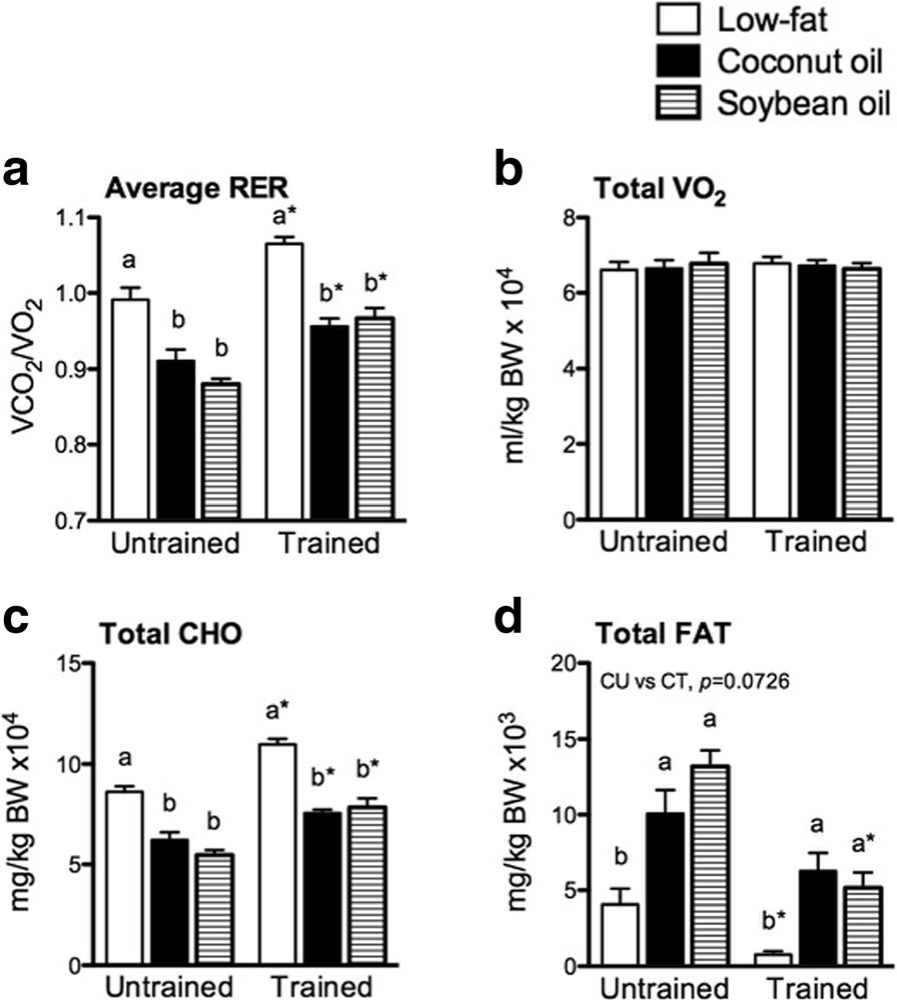
Basal indirect calorimetry. **a** Average RER, (**b**) total VO_2_, (**c**) total CHO, and (**d**) total FAT of mice at rest during a 24h light-dark cycle. Values are presented as means ± SEM (*n* = 8). Significant differences among groups of different diets were determined by one-way ANOVA followed by Newman-Keuls post-hoc test. Dissimilar alphabets indicate significant differences (*p* < 0.05). Significant differences between groups of the same diet having different training states were determined by Student’s t-test (*, *p* < 0.05)

Indirect calorimetry at rest 24 h after the last training session was performed to determine the effects of diet and training on whole-body metabolism. Basal RER significantly increased in L relative to C and S in either training state (*p* < 0.05) (Fig. 1a). Furthermore, training increased RER in all diet groups (*p* < 0.05). Basal VO_2_ and energy expenditure (not shown) were not significantly affected by diet nor training (Fig. 1b). L in either training state increased carbohydrate oxidation (CHO) than C and S (*p* < 0.05) (Fig. 1c). Furthermore, training increased CHO in all diet groups (*p* < 0.05). Fat oxidation (FAT) in L was significantly decreased relative to C and S in either training state (*p* < 0.05) (Fig. 1d). Training decreased FAT in all diet groups but significance (*p* < 0.05) was observed only in L and S but not in C (*p* = 0.0726). Therefore, at rest in ad libitum fed state, training shifted substrate utilization favoring CHO beyond the effects of diet. Furthermore, coconut oil and soybean oil slightly but differently affected FAT with training.

### Training but not diet strongly influenced whole-body metabolism during exercise

**Fig. 2:**
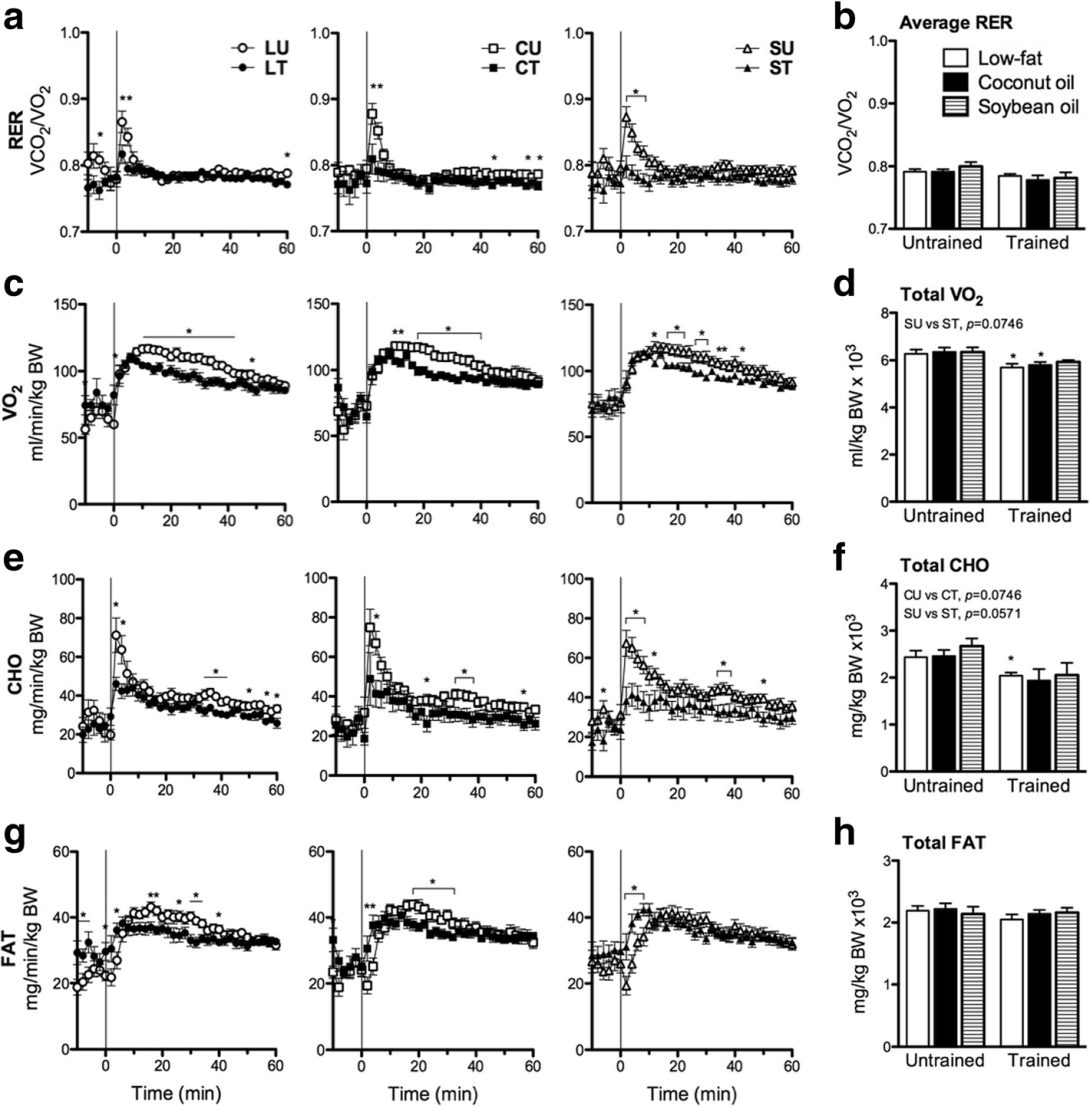
Exercise indirect calorimetry. **a**,**b** RER, (**c**,**d**) VO_2_, (**e**,**f**) CHO, and (**g**,**h**) FAT of mice during a 60min treadmill run presented as time-course changes (**a**,**c**,**e**,**g**) and average and total values (**b**,**d**,**f**,**h**). No significant difference in each time point and average and cumulative values was observed among groups of different diets as assessed by one-way ANOVA while significant differences were observed between groups of the same diet having different training status as assessed by Student’s unpaired t-test (*, *p* < 0.05). Values in both types of graph are presented as means ± SEM (*n* = 9-13)

Energy metabolism was measured during the first hour of endurance test. Diet groups had similar RER in either training state (Fig. 2a, b). Training decreased RER in several time points during the run especially at the onset of running (*p* < 0.05). Diet groups had similar VO_2_ in either training state (Fig. 2c, d). Training lowered VO_2_ at several points during the run (*p* < 0.05). However, cumulative data showed that S with training was not significantly different to its untrained counterpart (*p* = 0.0845). Diet groups had similar CHO in either training state (Fig. 2e, f). However, trained groups had consistently lower CHO during the run (*p* < 0.05). Furthermore, drastic elevation in CHO at the onset of running in untrained groups was absent with training. Cumulative CHO was lower with training but significant only in L (*p* < 0.05, *p* = 0.0746 and *p* = 0.0571 in L, C and S, respectively). Diet groups had similar FAT in either training state (Fig. 2g, h). FAT was higher in the trained groups particularly in the first few time points of the run (*p* < 0.05). Except in S, a reversal was observed as the run progressed with untrained groups having higher FAT than trained groups. In all parameters, no significant differences at any time point were observed among diet groups in either training state. Therefore, diet composition did not affect whole-body metabolism among groups of the same training status during exercise. On the other hand, training strongly affected exercise metabolism causing a slight shift to FAT if relative contribution to energy expenditure was considered as reflected in the mild but observable lowering of RER.

### Training overwhelmed the treadmill endurance improvement effect of coconut oil in the untrained state

**Fig. 3:**
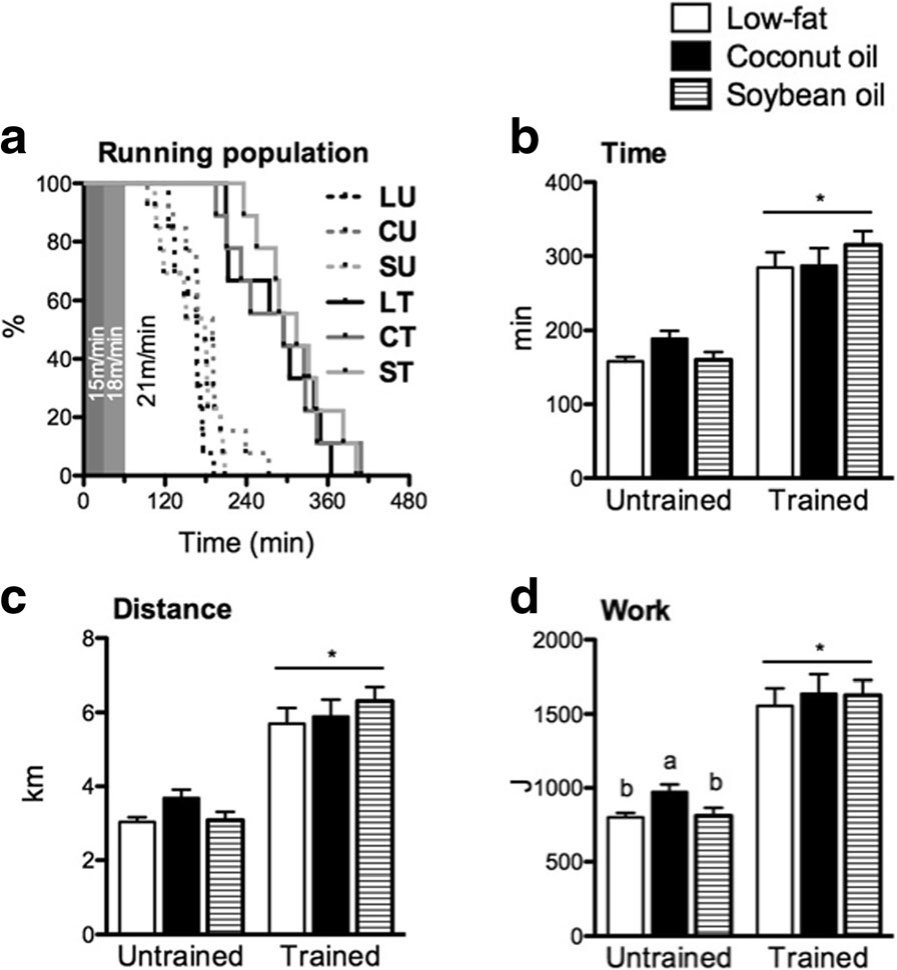
Treadmill endurance performance. **a** Running population plotted against time, (**b**) time-to-exhaustion, (**c**) distance and (**d**) work of mice subjected to running-to exhaustion test. Each point in A represents an individual mouse. For the bar graphs, data are presented as means ± SEM (*n* = 9-13). Significant differences among groups of different diets were determined by one-way ANOVA followed by Newman-Keuls post-hoc test. Dissimilar alphabets indicate significant differences (*p* < 0.05). Significant differences between groups of the same diet having different training states were determined by Student’s t-test (*, *p* < 0.05)

All mice were able to run past 60 min and exceed the intensity of 18 m/min (Fig. 3a). Diet groups had similar time-to-exhaustion and distance in either training state (Fig. 3b, c). Training increased to nearly double the treadmill endurance of untrained counterparts (*p* < 0.05) regardless of diet. Calculating for work revealed that C increased treadmill endurance than L and S in the untrained but not in the trained state (*p* < 0.05) (Fig. 3d). Therefore, coconut oil slightly but significantly improved endurance in the untrained state but did not potentiate the endurance improvement observed with training.

### Coconut oil negatively affected glycogen accumulation with training but promoted sparing of muscle glycogen during exercise

Pre- (basal) and post-exercise (30 min run) glucose-related metabolites were measured and presented in Table 3. Pre-exercise serum glucose was similar among diet groups in either training state. Training tended to decrease serum glucose. At post-exercise, all groups had significantly increased serum glucose (*p* < 0.05). L tended to decrease serum glucose than C and S and this was highlighted in trained groups (*p* < 0.05). Pre-exercise muscle glycogen was similar among diet groups in either training state. However, training increased this in L and S but not in C (*p* < 0.05). At post-exercise in the untrained state, it decreased (*p* < 0.05) relative to pre-exercise values in L and C while a non-significant decrease (*p* = 0.0672) was observed in S. With training, muscle glycogen also significantly decreased in L and S (*p* < 0.05) but not in C. Pre-exercise liver glycogen was similar among diet groups in the untrained state. It was significantly lower in C than L in the trained state (*p* < 0.05). Also, a non-significant decrease (*p* = 0.0735) was observed in C with training relative to its untrained counterpart. However, a longer run might be required to observe utilization of glycogen in the liver. Therefore, coconut oil prevented glycogen accumulation in the muscle and liver with training but preserved it in the muscle during exercise.

**Table 3:** Pre- and post-exercise metabolites

Training		Untrained (*n* = 8/group)			Trained (*n* = 8/group)		
Diets		Low-fat	Coconut oil	Soybean oil	Low-fat	Coconut oil	Soybean oil
Serum glucose, mg/dL	Pre-	152.9 ± 12.7	156.1 ± 8.3	156.0 ± 5.1	131.1 ± 5.8	142.7 ± 6.6	146.1 ± 5.5
	Post-	203.1 ± 6.0^†^	219.6 ± 10.0^†^	219.6 ± 8.2^†^	177.9 ± 8.1^b†^	194.5 ± 5.0^ab†^	202.6 ± 3.4^a†^
	%∆	32.83	40.68	40.77	35.70	36.30	38.67
Muscle glycogen, mg/g	Pre-	1.548 ± 0.199	1.518 ± 0.193	1.606 ± 0.199	2.154 ± 0.175*	1.703 ± 0.208	2.294 ± 0.173*
	Post-	0.932 ± 0.156^†^	0.942 ± 0.125^†^	1.083 ± 0.173^1^	0.764 ± 0.080^b†^	1.335 ± 0.147^a^	1.182 ± 0.229^ab†^
	%∆	−39.80	−37.98	−32.57	− 64.54	−21.61	−48.47
Liver glycogen, mg/g	Pre-	71.06 ± 3.87	59.93 ± 4.78	64.28 ± 9.09	63.13 ± 2.67^a^	46.42 ± 5.09^b2^	54.73 ± 5.15^ab^
	Post-	79.87 ± 5.74^a^	56.25 ± 4.41^b^	73.61 ± 3.08^a^	70.57 ± 5.78	56.43 ± 5.73	56.76 ± 2.56
	%∆	12.40	−6.14	14.51	11.79	21.56	3.71
Serum TG, mg/dL	Pre-	118.0 ± 10.2	116.3 ± 10.7	107.3 ± 8.5	109.4 ± 10.4	115.4 ± 6.5	115.4 ± 16.1
	Post-	101.8 ± 9.0^a^	93.3 ± 5.3^ab^	76.3 ± 4.7^b†^	87.8 ± 5.8	102.7 ± 14.7	71.0 ± 4.0^†^
	%∆	− 13.73	− 19.77	−28.91	−19.71	− 11.01	−38.44
Serum NEFA, mEq/L	Pre-	0.878 ± 0.078	0.817 ± 0.074	0.660 ± 0.030	0.652 ± 0.072*	0.794 ± 0.061	0.674 ± 0.054
	Post-	0.738 ± 0.040^a^	0.675 ± 0.096^a1^	0.550 ± 0.014^b†^	0.646 ± 0.037	0.656 ± 0.036^1^	0.551 ± 0.021^1^
	%∆	−15.93	− 17.45	−16.64	−0.86	− 17.31	−18.18
Serum β-HB, μmol/L	Pre-	24.22 ± 4.35	20.56 ± 2.59	24.06 ± 6.73	12.73 ± 2.10*	17.87 ± 2.93	15.75 ± 2.18
	Post-	170.10 ± 34.82^†^	257.50 ± 22.18^†^	178.60 ± 29.39^†^	96.90 ± 18.08^b†^	204.50 ± 24.61^a†^	135.60 ± 19.10^b†^
	%∆	602.31	1152.43	642.31	661.19	1044.38	760.95
Muscle TG, mg/g	Pre-	3.379 ± 1.002	3.133 ± 0.507	2.759 ± 0.602	2.320 ± 0.339	2.537 ± 0.677	2.894 ± 0.472
	Post-	1.527 ± 0.175	1.982 ± 0.317	2.111 ± 0.303	1.323 ± 0.324^1^	0.918 ± 0.190^†^	1.345 ± 0.236^†^
	%∆	− 54.81	−36.74	−23.49	− 42.97	−63.81	− 53.52

Pre-: pre-exercise (basal)

Post-: post-exercise (after 30 min run)

%∆: % difference between pre- and post- values

^a,b^: dissimilar alphabets indicate significant difference (*p <* 0.05) among groups of the same training status as assessed by one-way ANOVA followed by Newman-Keuls post-hoc test

*: *p* < 0.05 vs untrained counterpart as assessed by Student’s t-test

^†^: *p* < 0.05 vs pre- value as assessed by Student’s t-test

^1^: *p* < 0.1 vs pre- value as assessed by Student’s t-test

^2^: *p* < 0.1 vs untrained counterpart as assessed by Student’s t-test

### Coconut oil but not soybean oil promoted ketosis during exercise

Lipid- and ketone body-related metabolites at pre- and post-exercise are also presented in Table 3. Pre-exercise serum TG and NEFA was similar among diet groups in either training state. However, training caused significant decrease in NEFA in L (*p* < 0.05). At post-exercise, TG significantly decreased in S in both training states relative to pre-exercise values (*p* < 0.05). Post-exercise NEFA also decreased significantly in S without training (*p* < 0.05) and non-significantly in other C and S groups regardless of training (*p* < 0.1). Pre-exercise serum β-HB was similar among diet groups in either training state. However, it decreased with training and was significant in L (*p* < 0.05). At post-exercise, drastic elevation was observed in C in both training states and was emphasized by training relative to L and S (*p* < 0.05). Similarly, training relatively lowered β-HB levels post-exercise in all diet groups. Pre-exercise muscle TG was not significantly affected by diets nor training. Significant decrease was not observed in any untrained group at post-exercise. With training, muscle TG non-significantly decreased (*p* = 0.0522) in L and significantly decreased (*p* < 0.05) in C and S relative to pre-exercise values. Therefore, coconut oil caused ketosis during exercise but not at rest. On the other hand, training promoted intramuscular TG utilization during exercise.

### Mitochondrial enzyme activities varied with diets but were amplified by exercise

**Fig. 4:**
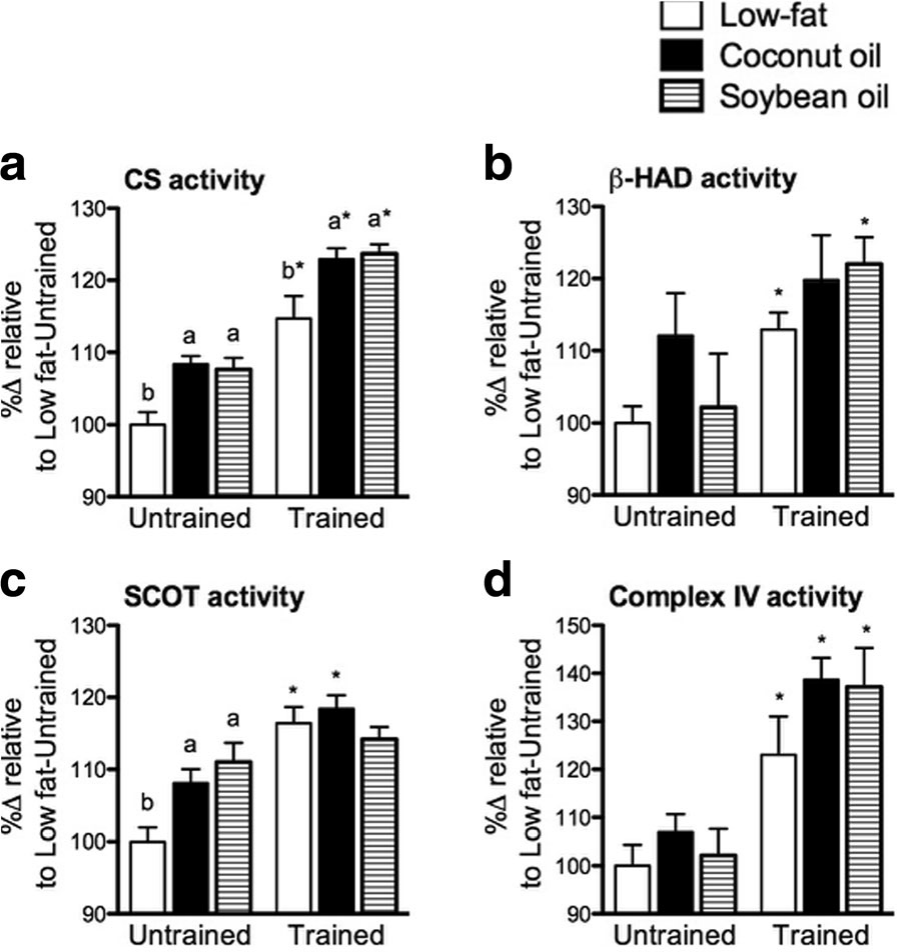
Muscle mitochondrial enzyme activities. **a** CS, (**b**) β-HAD, (**c**) SCOT, and (**d**) mitochondrial Complex IV activities of gastrocnemius from untrained and trained mice. Data are presented as means ± SEM (*n* = 8). Significant differences among groups of different diets were determined by one-way ANOVA followed by Newman-Keuls post-hoc test. Dissimilar alphabets indicate significant differences (*p* < 0.05). Significant differences between groups of the same diet having different training states were determined by Student’s t-test (*, *p* < 0.05)

The effects of diets and exercise on mitochondrial enzyme activities of the muscle were assessed. CS catalyzes the formation of citrate from oxaloacetate and acetyl-CoA and is commonly used as a measure of mitochondrial density in muscle [35]. In either training state, CS activity increased in C and S relative to L (*p* < 0.05) (Fig. 4a). Training significantly increased CS activity in all diet groups (*p* < 0.05). β-HAD activity reflects the capacity of cells to oxidize fatty acid [28]. In the untrained state, C had a non-significant increase in β-HAD relative to L and S. β-HAD activity was similar among diet groups in the trained state (Fig. 4b). Also, training significantly increased β-HAD activity in L and S (*p* < 0.05). An increase in β-HAD activity was also observed in C but this was not statistically significant. SCOT is an enzyme involved in ketolysis in the muscle [36]. In the untrained state, SCOT activity increased in C and S relative to L (*p* < 0.05) (Fig. 4c). Significant difference among diet groups in SCOT activity was not observed with training. Training increased SCOT activity in all groups but significant only in L and C (*p* < 0.05). Complex IV catalyzes the last step of the electron transport chain in the mitochondria and also regulates oxidative phosphorylation [37]. Similar complex IV activity was observed among diet groups in either training state (Fig. 4d) and training increased its activity (*p* < 0.05). Overall, coconut oil and soybean oil increased some muscle mitochondrial enzyme activities in the untrained state. However, training increased and normalized these muscle mitochondrial enzyme activities across the diet groups.

### Coconut oil inhibited training-induced increases in mRNA expression of PPARβ/δ and some target genes in the muscle

**Fig. 5:**
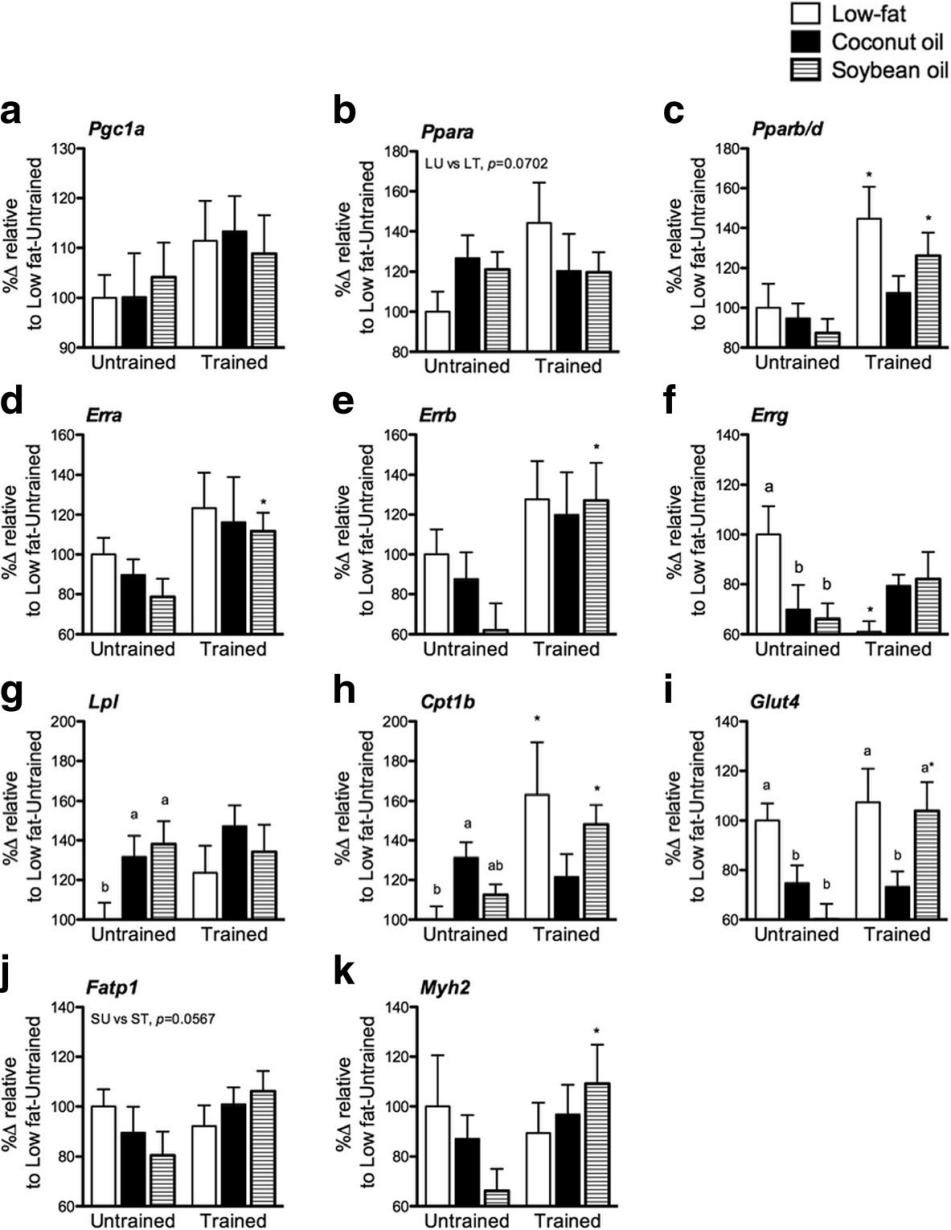
Muscle mRNA expression. **a** *Pgc1a*, (**b**) *Ppara*, (**c**) *Pparb/d*, (**d**) *Erra,* (**e**) *Errb,* (**f**) *Errg,* (**g**) *Lpl,* (**h**) *Cpt1b,* (**i**) *Glut4,* (**j**) *Fatp1,* and (**k**) *Myh2* expression in gastrocnemius from untrained and trained mice. Data are presented as means ± SEM (*n* = 8). Significant differences among groups of different diets were determined by one-way ANOVA followed by Newman-Keuls post-hoc test. Dissimilar alphabets indicate significant differences (*p* < 0.05). Significant differences between groups of the same diet having different training states were determined by Student’s t-test (*, *p* < 0.05)

Changes in mitochondrial enzyme activities and whole-body metabolism are influenced by transcriptional adaptations in the muscle. We measured known transcription factors affecting the said processes. Diet and training failed to significantly influence *Pgc1a* (Fig. 5a). *Ppara* non-significantly increased in C and S relative to L (Fig. 5b). While training failed to increase *Ppara* in C and S, 40% increase albeit not significant (*p =* 0.0702) was observed in L. *Pparb/d* expression was not affected in untrained diet groups (Fig. 5c). Training increased *Pparb/d* expression in L and S but not in C (*p <* 0.05). ERRs showed relative inhibition of expression in C and S in the untrained state (Fig. 5d-f). Significant decrease in expression was observed only in *Errg* (*p <* 0.05). Training increased the expression of *Erra* and *Errb* in all groups but significant only in S (*p <* 0.05). On the other hand, *Errg* decreased with training in L while significant increase was not observed in C and S (*p <* 0.05).

We also determined the expression of some PPAR targets. In the untrained state, *Lpl* expression was significantly increased in C and S relative to L (*p* < 0.05) (Fig. 5g). With training, the intergroup differences were abolished which could be attributed to the non-significant increase (20%) in L. In the untrained state, *Cpt1b* was significantly increased in C relative to L but not to S (*p* < 0.05) (Fig. 5h). With training, *Cpt1b* did not increase in C in contrast to considerable and significant (> 50%; *p* < 0.05) increases in L and S. *Glut4* was significantly decreased in C and S relative to L (*p* < 0.05) (Fig. 5i). Training did not change its expression in L and C. In the S however, significant increase similar to the level of L was observed (*p* < 0.05). *Fatp1* expression was similar among diet groups in either training state. Training caused a non-significant increase (*p* = 0.0567) in S but not in L and C (Fig. 5j). *Myh2* non-significantly decreased in S in the untrained state (Fig. 5k). Training significantly increased *Myh2* expression and normalized its expression with L and C (*p* < 0.05). Lastly, no significant differences in *Cd36* and *Pdk4* were observed because of diet nor training (not shown).

In summary, the amount of fat but not the type significantly affected the expression of PPARs and ERRs in the untrained state. With training, however, the responses of these genes and their targets varied. PPARs were influenced by the type of fat while the ERRs with the exception of ERRγ were unaffected by diet. In particular, coconut oil inhibited increases in PPARβ/δ mRNA expression as well as some of its targets.

### Coconut oil inhibited training-induced significant increases in GLUT4 protein

**Fig. 6:**
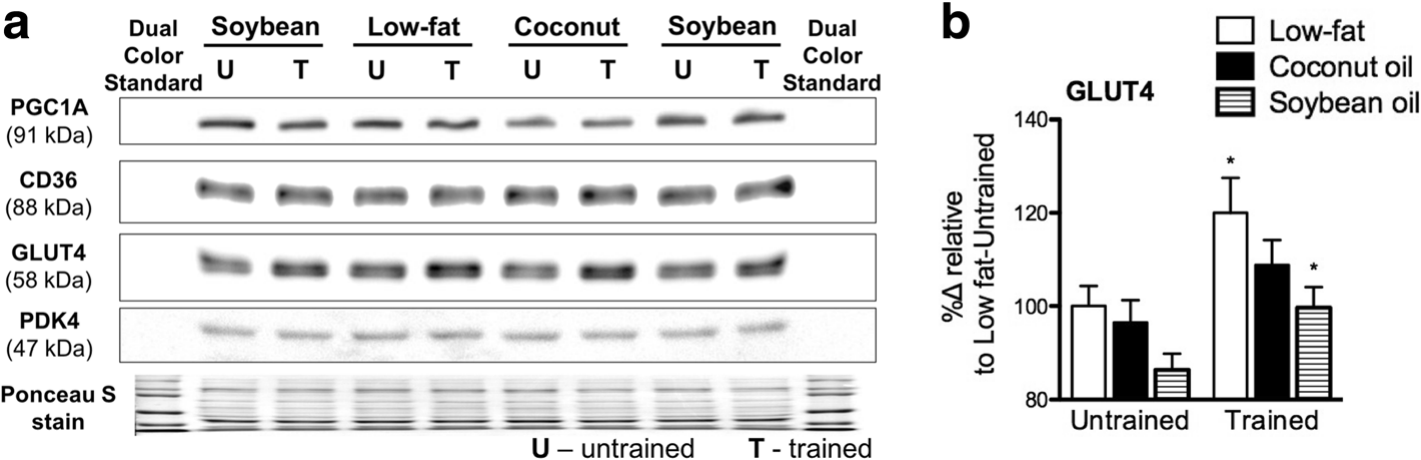
Muscle protein expression. **a** Representative immunoblot along with corresponding Ponceau S stained membrane, and (**b**) quantified GLUT4 protein expression in gastrocnemius from untrained and trained mice. Signals from proteins of interest were compared to total Ponceau S signal. Data are presented as means ± SEM (*n* = 8). No significant differences among groups of different diets were observed as determined by one-way ANOVA. Significant differences between groups of the same diet having different training states were determined by Student’s t-test (*, *p* < 0.05). Samples were processed simultaneously and each group was represented in each gel. Location and molecular weight of proteins were confirmed by visibly colored protein standards (invisible to chemiluminescent detection). Results were data pooled from 6 gels

Protein expression of glucose and fatty acid metabolism-related genes in the muscle were measured by immunoblotting. GLUT4 protein expression was non-significantly decreased in S in the untrained state (Fig. 6a, b). Training increased GLUT4 protein in all diet groups but significant only in L and S (*p* < 0.05) but not in C (*p* > 0.1). Protein expression of PGC1A, CD36, and PDK4 were not significantly influenced by diet nor training (Fig. 6a; quantification not shown). Therefore, training increased GLUT4 protein but coconut oil prevented the same degree of induction as in other diets.

### Coconut oil and soybean oil differently affected liver mitochondrial enzyme activation with training

**Fig. 7:**
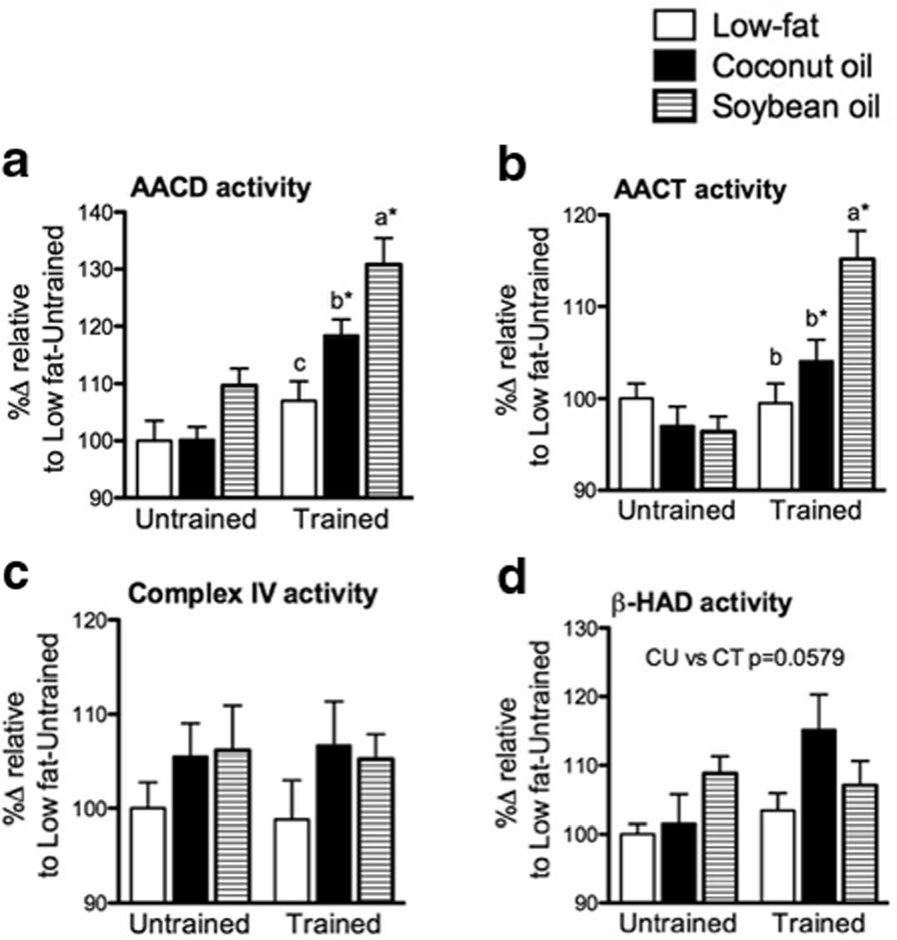
Liver mitochondrial enzyme activities. **a** AACD, (**b**) AACT, (**c**) Complex IV and (**d**) β-HAD activities of liver from untrained and trained mice. Data are presented as means ± SEM (*n* = 8). Significant differences among groups of different diets were determined by one-way ANOVA followed by Newman-Keuls post-hoc test. Dissimilar alphabets indicate significant differences (*p* < 0.05). Significant differences between groups of the same diet having different training states were determined by Student’s t-test (*, *p* < 0.05)

The effects of diets and training on mitochondrial functions of the liver were assessed. AACT and AACD catalyze the reversible conversion of acetyl-CoA to acetoacetyl-CoA and the non-reversible conversion of acetoacetyl-CoA to acetoacetate, respectively [33]. AACT and AACD activities were significantly different among diet groups with training but not in the untrained state (Fig. 7a, b). AACT significantly increased in S than L and C (*p* < 0.05). AACD, on the other hand, was significantly higher in S than C and was significantly higher in C than L (*p* < 0.05). Similar to AACT, training significantly increased AACD activity in C and S but not in L (*p* < 0.05). Complex IV activity was not significantly affected by diet nor training but a trend towards increased activity could be observed in C and S in both training states (Fig. 7c). β-HAD was similar among diet groups in either training state (Fig. 7d). However, there was a robust but non-significant (*p* = 0.0579) increase in β-HAD activity with training in C. Therefore, in the liver, coconut oil tended to improve β-oxidation with moderate ketogenic enzyme activation while soybean oil strongly activated ketogenic enzymes but not β-oxidation with training. However, increased mitochondrial density was not observed as universal changes in mitochondrial functions were absent.

### Coconut oil but not soybean oil promoted PPARα mRNA expression in the liver with training

**Fig. 8:**
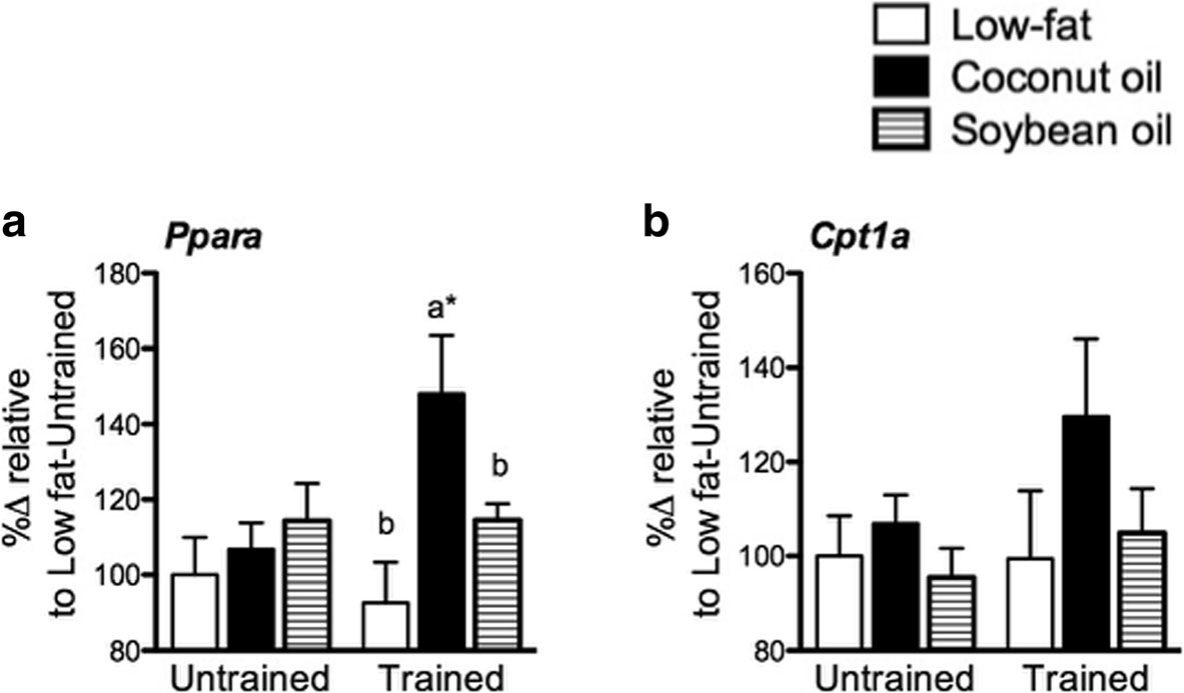
Liver mRNA expression. **a** *Ppara*, and (**b**) *Cpt1a* expression in liver from untrained and trained mice. Data are presented as means ± SEM (*n* = 8). Significant differences among groups of different diets were determined by one-way ANOVA followed by Newman-Keuls post-hoc test. Dissimilar alphabets indicate significant differences (*p* < 0.05). Significant differences between groups of the same diet having different training states were determined by Student’s t-test (*, *p* < 0.05)

PPARα influences hepatic ketogenesis and β-oxidation [10]. *Ppara* in the liver was similar among diet groups in the untrained state (Fig. 8a). Training significantly increased *Ppara* in C relative to L and S, and to its untrained counterpart (*p* < 0.05). *Cpt1a* non-significantly increased with training in C (Fig. 8b) while *Hmgcs2* was not significantly affected by diet nor training (not shown). Therefore, in the untrained state, the type of fat did not affect PPARα gene expression in the liver while coconut oil but not soybean oil induced its expression with training.

## Discussion

Fat as a bioactive compound influences metabolism by inducing muscle and liver phenotypic remodeling through transcriptional activation of PPARs [15, 17, 38–40]. We hypothesized that diets varying in fat source and proportion together with training would lead to different adaptations in the muscle and liver consequently affecting whole-body metabolism and endurance. Comparative studies on the effects of different diets with training have been conducted [20, 41, 42]. However, data on energy expenditure at rest and during exercise, substrate utilization, and gene transcription are scarce. Coconut oil is a good source of MCFAs which are rapidly metabolized relative to LCFAs [43]. MCFAs with training improve endurance in swimming [20] but other aspects of adaptation required further investigation. We aimed to update the current knowledge on the effects of MCFAs and fat types. We show that fat source and content in the diet exert variably influence different aspects of basal and treadmill training adaptations particularly on endurance, exercise whole-body metabolism, energy substrate storage and utilization, and genetic and biochemical characteristics of the muscle and liver.

Medium-fat diets increased FAT regardless of fat type and training promoted CHO at rest without affecting energy expenditure. Utilizing a lower intensity training protocol with the same soybean oil diet increased CHO, but it accompanied increased energy expenditure without affecting FAT [23] suggesting that different training intensities differentially affect resting metabolism [44, 45]. Our data in relation to [15] suggest that higher absolute MCFAs content may increase VO_2_ even without training relative to LCFAs.

Whole-body metabolism during exercise under different diets is associated with changes in VO_2_max [42]. Unfortunately, we could not measure VO_2_max because of technical limitations. During exercise under slight food deprivation, training but not diet influenced whole-body metabolism suggesting that at rest with ad libitum feeding, diet composition determined differences in resting energy metabolism while general effects were due to training. Moreover, training lowered VO_2_ (and energy expenditure) implying that exercise economy increased in trained groups during exercise [46]. In our previous study using a lower training intensity, decreased RER without changes in VO_2_ was observed suggesting higher fat utilization [23]. These observations further indicate that changes in energy metabolism at rest or during exercise is influenced by training intensity. It is important to note that inaccurate calculations in CHO and FAT, especially in C with ketosis during exercise, may exist because the complete oxidation of acetoacetate and β-HB give respiratory quotient values of 1.0 and 0.89, respectively [47]. Unfortunately, corrections for ketone body oxidation and its relative contribution to substrate metabolism could not be performed because a time-course ketone body profile in the blood was not available.

Our group and others show that diets high in fat induce muscle mitochondrial biogenesis and also imply that MCFAs promote mitochondrial biogenesis better than LCFAs at the same absolute concentration [15, 17]. β-HAD activity increased in C without training. Also, in C2C12 muscle cells, C10 and C12 fatty acids increased succinate dehydrogenase activity [15]. However, changes in the activities of other enzymes may not be entirely dependent on fatty acid species but on their amount as seen with SCOT and CS. We also showed that training universally increased mitochondrial function suggesting mitochondrial biogenesis. Therefore, elevated oxidative capacity in C, albeit small, likely improved endurance in the untrained state as measured by work while robust increase in mitochondria improved endurance in trained mice regardless of diet [48, 49]. In contrast with the swimming modality, MCFAs increased swimming time attributed to increased CS and SCOT relative to LCFAs [20] underscoring the notion that different training modalities variably influence adaptation, and potentially, endurance.

Exercise increases PGC1A and this co-activates or potentiates the PPARs and ERRs in the control of mitochondrial biogenesis [9]. We did not observe changes in PGC1A mRNA and protein despite increased mitochondrial function suggesting differential effects of training intensity on their half-lives [23, 50, 51]. However, we show that diets influenced basal and training-induced changes in mRNA expression of PPARs and ERRs. ERRs had decreased expression in medium-fat diets. This did not negatively affect mitochondrial enzyme activities, *Pgc1a* or *Erra* in the untrained state suggesting that at the basal level, homeostatic control and/or other ERR isotypes likely compensated for decreased mRNA expression of ERRγ [52, 53]. Training increased *Erra* and *Errb,* which could explain the increased mitochondrial biogenesis in the muscle [49].

High-fat diets increases *Ppara* but not the other isotypes in rats [54]. We did not observe significant elevations in *Ppara* in the untrained state possibly due to a relatively lower fat content of our diets. On the other hand, only *Pparb/d* increased with training in contrast to increased *Ppara* and unchanged *Pparb/d* when trained at a lower intensity even with the same soybean oil diet [23] suggesting that PPARs respond differently with training intensity. Interestingly, coconut oil impaired the training-induced upregulation of *Pparb/d*. Consistent with increased *Pparb/d* with training, target genes related to glucose and fat utilization, and fiber type remodelling (*Glut4*, *Cpt1b*, *Fatp1* and *Myh2*) responded similarly especially in S [55–58]. Because exercise increases fatty acid uptake by translocation of CD36 to the sarcolemma, not only increased expression of PPARβ/δ but also increased fatty acid-induced activation and availability could upregulate these targets [23, 59] which may explain some of the differences between S and L with training. On the other hand, these changes could not be attributed to increased CD36 as its mRNA and protein did not respond as expected [23] indicating that training intensity affects specific genetic adaptations.

The expression of *Cpt1b* and *Lpl* with medium-fat diets in the untrained state is probably related to PPARα as this isotype also controls their transcription in the muscle [60]. Because functions of PPAR isotypes overlap in some of these genes, we could not discount the contribution of PPARγ. Although PPARγ is abundant in adipose tissues, it is also present in skeletal muscle and MCFAs and LCFAs strongly activate PPARγ [11–14, 61].

Total caloric intake was similar among diet groups. This means that the minimum amount of consumed soybean oil was similar among diet groups suggesting that coconut oil inhibited training-induced upregulation of PPARβ/δ and some downstream targets. Whether MCFAs inhibited LCFAs by competitive binding in PPARβ/δ activation requires further research. While competitive binding assays between fatty acids and synthetic agonists have been performed [11, 12, 14], competitive binding assay among fatty acids to PPARs has yet to be undertaken. Overall, PPAR-related gene transcription as a training adaptation was influenced by the type of fat in the diet. Also, these adaptations reflect the route of catabolism of energy substrates within these diets during exercise.

In the untrained state, diets did not affect pre-exercise muscle glycogen possibly due to similar circulating lipids or serum β-HB as these influence glycogen storage [62–65]. Training increases muscle glycogen but ketone bodies, particularly acetoacetate, inhibit insulin-stimulated glucose uptake that occurs during feeding after training [66]. This may explain the inhibited glycogen accumulation in C. Furthermore, while GLUT4 is not essential for glycogen repletion per se it could influence glycogen accumulation with insulin post-exercise by increasing the rate of glucose transport [67–69]. Liver glycogen accumulation was also impaired in C and to a lesser extent in S which was emphasized by training. This could be partly explained by exhaustion of hepatic glycogen reserves with MCFAs and the glycogen replenishing effect carbohydrates [70, 71].

Increased muscle glycogen and glycogen sparing improves endurance by slowing the utilization of circulating glucose and liver glycogen [72, 73]. Muscle glycogen is spared by improved utilization of fatty acids and ketone bodies [74–77] linking glycogen sparing with high serum β-HB, decreased serum NEFA and intramuscular TG especially in C with training post-exercise. These suggest the existence of compensatory mechanisms to preserve endurance despite low pre-exercise muscle glycogen in this group. Nevertheless, muscle glycogen availability and utilization together with increased oxidative and mitochondrial functions likely promoted robust endurance improvement in all trained groups.

In the liver, unlike LCFAs, MCFAs can bypass fatty acid transport proteins to enter the cell and mitochondria for oxidation and these undergo β-oxidation for complete oxidation or ketogenesis [36, 59, 78–81]. Improved oxidative capacity with increased upstream β-HAD activity suggested increased capacity to produce ketones particularly in C [82] despite higher downstream ketogenic enzyme activities in S than C. Furthermore, because MCFAs are undetectable in the serum at rest, the overwhelming increase in β-HB during exercise in C suggests that liver and adipose tissues stored MCFAs, released them to circulation during exercise and were immediately catabolized [6, 20, 79, 83–85].

Fatty acid oxidation and ketogenesis in the liver is controlled by PPARα [10]. Diets did not influence *Ppara* but training increased its expression in C. Fibroblast growth factor 21 (FGF21) is induced by acetoacetate via an upstream regulator and this upregulates PPARα [38, 86] thus connecting the link between training, coconut oil, increased ketone bodies during training, and increased *Ppara*. While non-significant, increased *Cpt1a* and β-HAD activity in this group suggest PPARα activation in the liver [82]. *Hmgcs2*, the gene that encodes the first enzyme of ketogenesis [36], was unaffected by diet nor training suggesting that high β-HB observed post-exercise in C was primarily caused by increased supply of ketogenic precursors for β-oxidation in C rather than changes in ketogenic activity. On the other hand, relatively lower β-HB with training at post-exercise is likely because of increased muscular utilization accompanying increased SCOT activity. Whether increased oxidative capacity prevented increase of liver weight in C with training was not investigated. Overall, coconut oil with training promoted liver remodeling to an oxidative phenotype without influencing mitochondrial biogenesis.

## Conclusion

Our study provides evidence that prolonged feeding of coconut oil, and indirectly MCFAs, can improve endurance even in the untrained state. More importantly, training dictates endurance in wild-type mice [23]. Moreover, while training increases mitochondrial functions in the muscle, training-induced transcriptional adaptations in the muscle and liver are differentially influenced by diet. Our data suggest that with training coconut oil inhibits PPARβ/δ in the muscle while activating PPARα in the liver. This study and that of Fushiki, et al. and Manio, et al. [20, 23] highlight the importance of the modality used in training and endurance testing as these clearly influence specific training adaptations. Also, we show that results of exercise studies depend on diet composition and this has to be carefully considered in data interpretation. Unlike most studies investigating the effects of fat, the caloric composition of our medium-fat diets is lower and does not greatly differ from the diets of athletes [87] which may explain why observed changes were relatively modest. Although these adaptations benefit endurance exercise, activities relying on anaerobic glycolysis may be impaired by coconut oil due to reduced glycogen storage.

## Abbreviations

β-HAD: β-hydroxyacyl-CoA dehydrogenase
β-HB: β-hydroxybutyrate
AACD: Acetoacetyl-CoA deacylase
AACT: Acetoacetyl-CoA thiolase
AMPK: AMP-activated protein kinase
ANOVA: Analysis of variance
C: Coconut oil diet
CD36: Fatty acid translocase/cluster of differentiation 36
CHO: Carbohydrate oxidation
CS: Citrate synthase
ERR: Estrogen-related receptor
FAT: Fat oxidation
GLUT4: Glucose transporter 4
L: Low-fat diet
LCFA: Long chain fatty acid
MCFA: Medium chain fatty acid
NEFA: Non-esterified fatty acids
PDK4: Pyruvate dehydrogenase kinase 4
PGC1A: PPARγ Coactivator 1α
PPAR: Peroxisome proliferator activated receptor
RER: Respiratory exchange ratio
S: Soybean oil diet
SCOT: Succinyl-CoA:3-oxoacid CoA-transferase
T: Trained
TBST: Tris-buffered saline with 0.1% Tween-20
TG: Triglycerides
U: Untrained
VO_2_: Oxygen consumption
